# *Helicobacter pylori* eradication rates using clarithromycin and levofloxacin-based regimens in patients with previous COVID-19 treatment: a randomized clinical trial

**DOI:** 10.1186/s12879-023-07993-8

**Published:** 2023-01-20

**Authors:** Ahmed Kamal, Ramy Mohamed Ghazy, Dalia Sherief, Aliaa Ismail, Walid Ismail Ellakany

**Affiliations:** 1 Department of Internal Medicine, Hepatology Unit, Faculty of Medicine, Alexandria University, 1 Khartoum Square, Alexandria, 21131 Egypt; 2 Tropical Health Department, High Institute of Public Health, Alexandria University, Alexandria, Egypt; 3 Department of Clinical Pathology, Faculty of Medicine, Kafr elsheikh University, Kafr elsheikh, Egypt; 4 Department of Dermatology, Venereology and Andrology, Faculty of Medicine, Alexandria University, Alexandria, Egypt; 5 Department of Tropical Medicine, Faculty of Medicine, Alexandria University, Alexandria, Egypt

**Keywords:** *Helicobacter pylori*, Antimicrobial resistance, COVID-19

## Abstract

**Background:**

*Helicobacter pylori* (*H. pylori*) is affecting half of the globe. It is considered a main causative organism of chronic gastritis, peptic ulcer disease, and different gastric maliganacies. It has been also correlated to extraintestinal diseases, including refractory iron deficiency anaemia, vitamin B12 deficiency, and immune thrombocytopenic purpura. The misuse of antibiotics during the coronavirus diseases 2019 (COVID-19) pandemic time can affect *H. pylori* eradication rates. Our aim was to compare the efficacy of clarithromycin versus levofloxacin-based regimens for *H. pylori* treatment in naïve patients after the COVID-19 pandemic misuse of antibiotics.

**Methods:**

A total of 270 naïve *H. pylori* infected patients with previous treatment for COVID-19 more than 3 months before enrolment were recruited. Patients were randomized to receive either clarithromycin, esomeprazole, and amoxicillin, or levofloxacin, esomeprazole, and amoxicillin.

**Results:**

A total of 270 naïve *H. pylori* infected patients with previous treatment for COVID-19 more than 3 months before enrolment were included, 135 in each arm. In total, 19 patients in the clarithromycin group and 18 patients in the levofloxacin group stopped treatment after 2–4 days because of side effects or were lost for follow-up. Finally, 116 subjects in the clarithromycin group and 117 in the levofloxacin group were assessed. The eradication rates in intention to treat (ITT) and per protocol (PP) analyses were: group I, 55.56% and 64.66%; and Group II, 64.44% and 74.36% respectively (p = 0.11).

**Conclusion:**

As COVID-19 pandemic has moved forward fast, high resistance rates of *H. pylori* to both clarithromycin and levofloxacin were developed after less than two years from the start of the pandemic. Molecular & genetic testing is highly recommended to identify antimicrobial resistance patterns. Strategies to prevent antibiotic misuse in the treatment of COVID-19 are needed to prevent more antibiotic resistance.

*Trial Registration*: The trial was registered on Clinicaltrials.gov NCT05035186. Date of registration is 2-09-2021.

## Background

*Helicobacter pylori* (*H. pylori*) is a Gram-negative bacillus infection affecting half of the globe [1]. It is considered a main causative organism of chronic gastritis, peptic ulcer disease, and gastric carcinoma [2, 3]. It has been also correlated to extraintestinal diseases, including refractory iron deficiency anaemia, vitamin B12 deficiency, and immune thrombocytopenic purpura [4].

According to the American College of Gastroenterology (ACG) Clinical Guideline, *H. pylori* first-line treatment consists of Clarithromycin triple therapy including a proton-pump inhibitor (PPI), clarithromycin, and amoxicillin or metronidazole for 14 days. This regimen is applied in regions where *H. pylori* clarithromycin resistance is less than 15% and in patients with no previous history of macrolide exposure [2]. Another regimen is levofloxacin triple therapy [5]. The later can achieve higher eradication rates than clarithromycin-based regimens [6].

The main etiologies for the failure of anti *H. pylori* treatment are low compliance [7] and antibiotic resistance [8]. Outpatient misuse of antibiotics resulted in a high rate of clarithromycin resistance and so the empirical use of clarithromycin in standard anti *H. pylori* regimens is not encouraged in many communities. The knowledge about the community use of antibiotics may be used as a tool to adapt treatment strategies and to predict susceptibility [9].

Azithromycin was suggested to be a beneficial drug against coronavirus disease of 2019 (COVID-19), due to its antiviral, anti-inflammatory properties and to prevent secondary bacterial infection [10]. Furthermore, azithromycin can reduce the levels of proinflammatory cytokines, including interleukin-6 (IL-6), which was suggested to reduce the severe acute respiratory syndrome coronavirus-2 (SARS-CoV-2) infection triggered cytokine storm and concomitant tissue damage [11].

Although clinical trials have explored that drugs like azithromycin, chloroquine, and ivermectin are ineffective against COVID-19, they are frequently prescribed by doctors and self-administered by the people in many world regions during the COVID-19 pandemic [12]. The use of antimicrobials against COVID-19 contributes to the increase in drug-resistant illnesses [12]. In Egypt, nearly 67%of Egyptian pharmacists said that patients who had any sign or symptom of COVID-19 infection were more likely to be given antibiotics, and 82%of medications were provided on physician recommendations. The principal antibiotics administered to patients suspected of having COVID-19 were azithromycin, ceftriaxone, linezolid, and levofloxacin. Azithromycin was administered to about 40% of individuals suspected of having mild to moderate symptoms while levofloxacin was administered to about 10% [13]. The vast use of azithromycin could lead to cross-resistance to other macrolides and hence affecting clarithromycin-based therapy for *H. pylori*. Although levofloxacin triple therapy can allow a better *H. pylori* eradication rate especially in cases of other antimicrobial resistance, the wide use of levofloxacin may change the global pattern of levofloxacin resistance [5].

The primary objective of this study was to address the efficacy of clarithromycin- and levofloxacin-based regimens as the first-line eradication therapy of *H. pylori* after the wide-scale misuse of antibiotics during the COVID-19 pandemic.

## Methods

### Study design

This open labelled randomized control trial study was conducted during the period from March 21, 2021, to September 30, 2021, recruiting patients from the outpatient clinics of Alexandria University hospitals, the largest hospital in Alexandria governorate that services also residents of the other 2 neighbouring Egyptian governorates, as well as those referred by clinicians working in inpatient and outpatient facilities. The report of this trial follows the recommendations of the Consort Statement for the quality of reports of parallel group, randomized trial.

### Sample size

Supposing the cure rate of the clarithromycin-based regimen and to the levofloxacin-based regimen is 69% versus 84.5% respectively, using Medcalc, the minimum required sample size was calculated as 116 patients for each arm (type 1 error = 5%, type II error = 20%). Each arm was increased by 10% to compensate for drop-out. The sample size was 135 for each arm. Two hundred seventy patients were enrolled.

Patients aged 18–65 years old with newly diagnosed *H. pylori* infection who were previously treated as having confirmed or suspected COVID-19 were included. The diagnosis was based on positive *H. pylori* stool antigen (HpSA, Perkin Elmer^®^, Bios, USA), urea breath test (Heliprobe^®^ Breath Card™, Kibion AB, Sweden), Rapid Urease test (Helicotec UT^®^ Plus, Strong Biotech Corporation, Taiwan), or detection of *H. pylori* during histopathological examination of gastric biopsies [14]. As per ACG clinical guidelines all patients with positive H.pylori test should be treated [2]. Test was done for those with peptic ulcer, history of peptic ulcer, presence or history of gastric malignancy, dyspepsia, those who need chronic usage of aspirin or analgesics and those who underwent endoscopy for upper GI symptoms [2]. The main presenting complaint in each patient was documented.

#### Group I

The first group received (amoxicillin 1 g/12 h, Clarithromycin 500 mg/12 h, esomeprazole 40 mg/12 h).

#### Group II

The second group received (esomeprazole 40 mg/12 h, levofloxacin 500 mg/24 h, and amoxicillin 1gm/12 h).

High doses of PPI were used for better eradication rates [15]. Patients were instructed to adhere to the drug regimen and were followed up for the possible side effects.

### Randomization

Computer based randomization was done in six-block increments. We chose a randomized design to avoid any accidental bias in group assignments.

### Blindness

Investigator and outcome assessor were blind while participants and care providers were not masked. Participants were unmasked to gain their confidence and so we could recruit more subjects. It was exceedingly difficult to mask care providers while participants were unmasked.

### Data collection

All patients were subjected to full history taking including demographic data and social history of smoking and alcohol consumption, thorough clinical examination, and laboratory investigations. Patient compliance was assessed by counting the remaining pills at pre-designed intervals. Patients with compliance of less than 80% were planned to be excluded from the study per-protocol (PP) analysis.

Patients were advised about the potential adverse events of the regimens investigated at the time of enrollment. All patients were requested to complete a questionnaire to report adverse reactions to the medication (diarrhea, taste disturbances, nausea, bloating, lack of appetite, vomiting, stomach discomfort, constipation, headache, and skin rash) [16]. Each symptom’s severity was scored from absence (0) to severe (3).

Owing to the rising rates of resistance to antimicrobials worldwide, all patients should have confirmation of eradication [17]. Consequently, after 6–8 weeks of the treatment period and at least 4 weeks after the end of antimicrobials and at least 2 weeks with no administration of PPIs, *H. pylori* eradication was assessed using the same detection test used for diagnosis. For those with a negative urea breath test and fecal H. pylori Ag before treatment but detectable H. Pylori after endoscopy, re-endoscopy was done to ensure eradication of *H. pylori*.

### Ethics

The study protocol got approval by the Ethical Committee of the Faculty of Medicine, Alexandria University, Egypt (Approval Number: 00012098) and the study was performed following the good clinical practice and the ethical principles for the medical research involving human subjects of the Declaration of Helsinki. Written informed consent was obtained from each participant.

### Statistical analysis

Statistical Analysis Both PP and ITT analyses were performed. Statistical analyses were performed using the computer program Statistical Package for the Social Sciences (SPSS), version 26.0 (IBM, Chicago, USA). The independent t-test was used for the comparison of 2 group means. The demographic data and frequencies of adverse reactions were compared using the chi-square test or Fisher’s exact test, when appropriate. The incidence of side effects was considered as a binomial variable (present-absent). Any “side effect” was considered absent if the subject reported the same complaint at baseline visit, as assessed by the questionnaire. Data were presented as the mean ± standard deviation or number and percentage. Differences were considered significant at p < 0.05. To detect differences in *H. pylori* eradication rates and the incidence of side effects, the χ^2^ and the Fisher exact tests were used. Odds ratio (OR) for achieving *H. pylori* eradication with 95% confidence intervals (95% CI) were calculated.

## Results

**Fig. 1:**
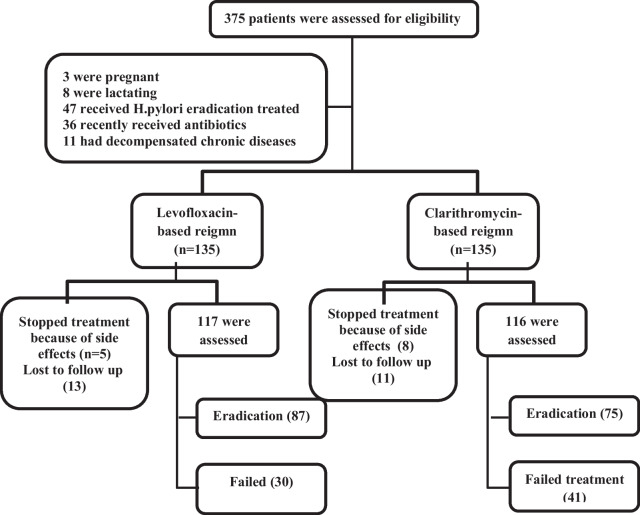
CONSORT flow chart of the study

In this study, 270 subjects were included, 135 in each arm. In total, 19 patients in the clarithromycin group and 18 patients in the levofloxacin group stopped treatment after 2–4 days because of side effects or were lost for follow-up before assessment of *H. pylori* eradication. Finally, 116 subjects in the clarithromycin group and 117 in the levofloxacin group were assessed. The CONSORT flow chart is shown in Fig. 1.

Participants mean age was 41.9 ± 13.0 years, 58.8% were males, 63.4% were married, 88.0% were living in urban areas, and 60.1% had no history of chronic diseases. All remaining patients had shown more than 80% compliance.

There was no statistically significant difference between the clarithromycin-based regimen and the levofloxacin-based regimen regarding baseline characteristics, the main presenting complaint and the type of the used diagnostic test as shown in (Table 1). About 25.5% of the studied patients were smokers while all of them reported no alcohol consumption.

**Table 1:** Population characteristics according to arm of treatment

Characteristics		Clarithromycin-based regimen (n = 116)	Levofloxacin-based regimen (n = 117)	Test statistics	*P*	
Sex						
Male (%)		74 (63.8)	64 (54.7)	2.00	0.350	
Female		42 (36.2)	53 (45.3)			
Age, years mean ± SD		41.31 ± 13.2	42.92 ± 12.77	0.95	0.290	
Marital status						
Married		71 (61.2)	78 (66.7)	1.59	0.663	
Single		45 (38.8)	39 (33.3)			
Residence						
Urban		103 (88.8)	99 (84.6)	0.88	0.350	
Rural		13 (11.2)	18 (15.4)			
Smoking						
Smokers		33 (28.4)	27 (23.1)	0.37	0.560	
Nonsmoker		83 (71.6)	90 (76.9)			
Medical history						
No chronic disease		70 (60.3)	68 (58.1)	3.70	0.590	
Chronic disease		46 (39.7)	49 (41.9)			
The most annoying C/O						
Heart burn		9 (7.8)	11 (9.4)	0.82	0.850	
Epigastric pain		83 (71.6)	86 (73.5)			
Vomiting		16 (13.8)	12 (10.3)			
Reflux laryngitis		8 (6.9)	8 (6.8)			
Detection method						
Urea breath test		72 (62.1)	45 (38.5)	0.15	0.479	
Stool antigen		43 (37.1)	29 (24.8)			
Endoscopy		31 (26.7)	13 (11.1)			
Treatment						
Treatment duration		13.94 ± 0.03	13.93 ± 0.03	0.10	0.920	

The overall response rate to *H. pylori* eradication was 69.53%. Based on the PP and ITT analyses, higher treatment response was observed among patients exposed to the levofloxacin-based regimen 74.36% and 64.44% compared to the clarithromycin-based regimen 64.66% and 55.56% respectively. However, these differences were not statistically significant (p = 0.11, and p = 0.14 respectively) (Table 2)

**Table 2:** Per protocol and intention to treat analyses in both groups

Analysis	Regimen	Eradication rate	OR (95% CI)	p
Per protocol	Clarithromycin group	64.66 (75/116)	0.63 (0.36–1.11)	0.11
	Levofloxacin group	74.36 (87/117)		
Intention to treat	Clarithromycin group	55.56 (75/135)	0.69 (0.42–1.13)	0.14
	levofloxacin group	64.44 (87/135)		

### Side effect profile

There were no statistically significant differences between either group regarding side effects as described in Table 3.

**Table 3:** Experienced side effects in both groups

Variable	Clarithromycin-based regimen (n = 116)	Levofloxacin-based regimen (n = 117)	χ^2^	*P*
Epigastric pain	20 (17.2)	16 (13.7)	0.56	0.452
Vomiting	6 (5.1)	5 (4.3)	0.05	0.751
Diarrhoea	6 (5.1)	4 (3.4)	0.41	0.523
Nausea	13 (11.2)	14 (12.3)	0.04	0.844
Bloating	8 (6.9)	5 (4.3)	0.73	0.392
Change in taste	17 (14.7)	14 (12.0)	0.12	0.731
Skin rash	1 (0.9)	1 (0.9)	0.0	1.000

## Discussion

In this work, we demonstrated that the levofloxacin-based regimen resulted in a 74.36% eradication rate while the clarithromycin-based regimen showed a 64.66% eradication rate based on PP. Lower eradication rates were reported based on the ITT analysis (64.44%) for levofloxacin-based regimen and 55.56% for clarithromycin-based regimen. Both regimens had an unacceptable rate of eradication. While the eradication rate using levofloxacin was higher than that of the clarithromycin-based regimen, the difference did not reach statistical significance. Moreover, the eradication rate in the levofloxacin group was lower than expected.

Clarithromycin-based regimen’s eradication rate was 84% in a study performed in Newyork over patients treated between 2011 and 2017 [18]. A Cochrane meta-analysis reported an 82% H.pylori eradication rate [19]. Another meta-analysis reported the global eradication rate with non-bisthmus-based triple therapy to be 81% [20]. Levofloxacin was superior to clarithromycin in a study performed in 2017 and 2018 with an eradication rate of 81% [21]. Also, this regimen achieved an eradication rate of 86% on ITT analysis and 92% on PP analysis in another study [22].

Previously Elantouny et al. [23] had concluded that levofloxacin-based triple therapy is the recommended treatment of *H. pylori* infection in countries like Egypt with high clarithromycin resistance. In this study which had been conducted in 2018 in Egypt, the eradication rate in the levofloxacin group was 85% and it was 69% in the clarithromycin group (p = 0.001). Importantly, it had been shown that 50% and 6.7% of the children in Egypt suffer from clarithromycin and levofloxacin resistance, respectively [24]. In comparison to our results, these may point to a rapid rise in levofloxacin resistance against clarithromycin resistance.

During the COVID-19 pandemic, drug repurposing of on-market FDA-approved drugs was suggested to be more efficient and cost-effective compared to de novo drug discovery [25]. The vagueness that surrounded the nature, sequence, and mechanism of infection and resistance of SARS-CoV-2 resulted in extensive use of different classes of drugs to treat this respiratory virus including several systemic antibiotics [26]. The International Severe Acute Respiratory Infection Consortium study reported that antibiotics are prescribed to 72% of hospitalized patients [27]. Bacterial superinfections are one of the leading causes of global mortality and represent one of the main challenges for healthcare professionals in COVID-19 patients [27]. However, bacterial co-infection was only identified in 3.5% and secondary bacterial infection in 15.5% of patients [26, 28, 29]. Despite the variable clinical presentation of COVID-19, respiratory manifestations are the most common. The similarity of these manifestations to that of community acquired pneumonia drives clinicians to empirically use broad-spectrum antibiotics in this viral disease. Consequently, many reports recently delineate the emergence of multidrug resistant bacteria during the COVID-19 pandemic [29, 30]. This will hinder the strategic use of antibiotics for many diseases in the near future. Having considered that the effective treatment of *H. pylori* is antibiotic dependent, bacteria resistance represents an already established obstacle for effective treatment for *H. pylori* infection [31].

It has been shown that antibiotic resistance could be acquired via different mechanisms. Azithromycin was suggested to have a special role for community treatment of suspected COVID-19 due to its antiviral, anti-inflammatory, and immunomodulatory properties. Given its safety profile, low cost and oral route of administration, Azithromycin is a frequently used antimicrobial agent during the pandemic [32, 33]. Cross-resistance between azithromycin and clarithromycin is well known [34]. Macrolide antibiotics interfere with protein synthesis by binding to 23s ribosomal RNA of 50s ribosomal subunit. The clinically significant mechanism by which *H. pylori* evades clarithromycin is a point mutation in domain V of the 23 S rRNA gene thus preventing drug binding [35].

Respiratory fluoroquinolones have also been recommended in the treatment of community-acquired pneumonia in COVID-19 patients. Because of their potential antiviral activity and immunomodulatory properties, the use of respiratory fluoroquinolones in the treatment of SARS-CoV-2 was suggested [36]. Quinolones halt DNA synthesis through inhibition of bacterial type II topoisomerase (DNA gyrase) and topoisomerase IV. The mechanism of bacterial evasion to quinolones is through mutation of DNA gyrase or topoisomerase IV; plasmid-mediated resistance and efflux systems that decrease intracellular drug level [36]. It is possible that the extensive use of fluoroquinolones to treat COVID-19 patients resulted in segregation of mutations that subsequently induced fluoroquinolone resistance. This may explain the rapid decline in the fluoroquinolone eradication rate in Egypt now compared to the pre-COVID-19 era.

Clarithromycin-based triple therapy is still considered a drug of choice to treat *H. pylori* infection in Egypt according to the Egyptian recommendations in 2018 and the choice of the regimen should depend also on age, co-morbidities, concomitant drugs, and previous exposure [37]. According to the Maastricht V/Florence consensus report, clarithromycin-based triple therapy is not recommended if the local clarithromycin resistance rate exceeds 15% [38]. However, levofloxacin resistance is also proceeding at a rising rate, which necessitates avoiding its use in a population whose resistance rate is higher than 15% [39]. Results of our study points to a more rise in levofloxacin resistance in the Egyptian community after the COVID-19 pandemic which may be due to the misuse of levofloxacin in the management of COVID-19. It was estimated that about 18% of adult Egyptian people had used antibiotics to treat themselves from COVID-19 symptoms without physician consultation [40].

Strengths in our study include that it is the first one to address the problem of increasing H.pylori resistance after the COVID-19 pandemic. Limitations of our study include that both patients and care providers were not blinded. H.Pylori detection tools were not uniform, but this was to enable us to enrol more patients to the study. To overcome this limitation, the same detection method was reused to assess the treatment outcome including reendoscopy if needed aiming for less bias. All used methods have high and comparable sensitivity, specificity, and accuracy with negative predictive values above 90% [41, 42]. It is suggested that there is direct relation between antibiotic resistance and the time-lapse from previous exposure. One of limitations in our study is that we did not take the exact time between COVID-19 treatment and enrolment into consideration. We recommend future studies to take this point into consideration and so we can explore if there is a relation between *H. pylori* resistance to the time from previous COVID-19 treatment. Another limitation is that we did not document which type of antibiotics was used by each patient as a part of COVID-19 treatment. We recommend considering this in future studies.

## Conclusion

Both regimens showed lower than accepted eradication rates among subjects who were previously treated from COVID-19. This should raise the alarm about the increase in antibiotic resistance among these persons and among the community as a whole. This rising resistance can adversely impact the costs of *H. pylori* treatment and increase the risk of *H. pylori* related diseases. Further studies enrolling a larger number of patients with molecular and genetic testing are needed to elucidate the exact mechanism of antibiotic resistance of H. pylori in such patients. These studies can help policymakers to define the best cost-effective protocol for *H. pylori* management in view of the rising antibiotic resistance.

## Data Availability

The datasets used and/or analysed during the current study are available from the corresponding author on reasonable request.

## Abbreviations

ACG: American College of Gastroenterology
COVID-19: Coronavirus diseases 2019
CI: Confidence intervals
*H. pylori*: *Helicobacter pylori*
IL-6: Interleukin-6
ITT: Intention to treat
OR: Odds ratio
PP: Per protocol
PPI: Proton-pump inhibitor
SARS-CoV-2: Severe acute respiratory syndrome-coronavirus-2
SPSS: Statistical Package for the Social Sciences
